# Prevention of *Staphylococcus aureus* biofilm formation by antibiotics in 96-Microtiter Well Plates and Drip Flow Reactors: critical factors influencing outcomes

**DOI:** 10.1038/srep43854

**Published:** 2017-03-02

**Authors:** Suvi Manner, Darla M. Goeres, Malena Skogman, Pia Vuorela, Adyary Fallarero

**Affiliations:** 1 Pharmaceutical Sciences Laboratory, Faculty of Science and Engineering, Abo Akademi University, BioCity, Artillerigatan 6A, FI-20520, Turku, Finland; 2 Center for Biofilm Engineering, Montana State University, Bozeman, MT 59717, USA; 3 Pharmaceutical Design and Discovery (PharmDD), Pharmaceutical Biology, Division of Pharmaceutical Biosciences, Faculty of Pharmacy, University of Helsinki, Viikinkaari 5E, P.O. Box 56, FI-00014 University of Helsinki, Finland

## Abstract

Biofilm formation leads to the failure of antimicrobial therapy. Thus, biofilm prevention is a desirable goal of antimicrobial research. In this study, the efficacy of antibiotics (doxycycline, oxacillin and rifampicin) in preventing *Staphylococcus aureus* biofilms was investigated using Microtiter Well Plates (MWP) and Drip Flow Reactors (DFR), two models characterized by the absence and the presence of a continuous flow of nutrients, respectively. Planktonic culture of *S. aureus* was exposed to antibiotics for one hour followed by 24 hours incubation with fresh nutrients in MWP or continuous flow of nutrients in DFR. The DFR grown biofilms were significantly more tolerant to the antibiotics than those grown in MWP without the continuous flow. The differences in log reductions (LR) between the two models could not be attributed to differences in the cell density, the planktonic inoculum concentration or the surface-area-to-volume ratios. However, eliminating the flow in the DFR significantly restored the antibiotic susceptibility. These findings demonstrate the importance of considering differences between experimental conditions in different model systems, particularly the flow of nutrients, when performing anti-biofilm efficacy evaluations. Biofilm antibiotic efficacy studies should be assessed using various models and more importantly, in a model mimicking conditions of its clinical application.

Bacterial biofilms are organized communities of bacteria embedded in a self-produced matrix of extracellular polymeric substances (EPS). They represent the predominant bacterial lifestyle in most natural environments^1^. Biofilms are increasingly associated with human infections, especially due to the rise in use of medical devices, such as catheters, implants and pacemakers^2^,^3^. The increased host immune system evasion as well as tolerance and resistance to antimicrobials displayed by biofilms lead to failure of conventional antimicrobial therapy. Thus, biofilms cause persistent infections characterized by increased morbidity and mortality^4^,^5^. *Staphylococcus aureus* is one of the most frequent causes of nosocomial and medical device-related biofilm infections^6^.

Various *in vitro* biofilm models have been developed for growing biofilms and evaluating antimicrobial treatments against them. In general, biofilm models can be divided into two groups: open (dynamic) or closed (batch) systems^7^. By definition, a dynamic reactor system has a continuous flow of fresh nutrients whereas in a classically defined batch reactor, fresh nutrients are only added at the beginning of the experiment. In biofilm research, reactors are often used as semi-batch systems, meaning the nutrients are replenished at various times in the growth of the biofilm. Although all dynamic models will have at least some fluid shear caused by the flow of nutrients over the biofilm, in some dynamic models, the fluid shear rates are dependent upon specific design features such as rotating baffles (Centers for Disease Control (CDC) reactor), drums (annual reactor) or blades (Constant depth film fermenter)^8^,^9^. In a batch reactor, fluid shear occurs when the contents are mixed by either placing the reactor in an environmental shaker or on a stir plate. The DFR is categorized as a plug flow reactor where the biofilm is grown under a laminar flow of nutrients close to the air/liquid interface^10^. The DFR has been utilized to grow biofilms that occur in oral cavity^11^, chronic wounds^12^ and catheters^13^ and it has been used as a model system in medically relevant efficacy testing of antimicrobials^11^,^14^. However, due to its low throughput and large operational volume, the DFR is not typically used for drug screening.

On the other hand, MWP are one of the most widely used model systems in biofilm research. When plates are placed on a shaker, biofilms are formed under low shear conditions but the amount of nutrients available and aeration is limited^7^. MWP are the most relevant and well suited model for drug screening because they enable for high throughput, are suitable for several bacterial species and require only a small volume of test samples^8^,^15^,^16^. Additionally, MWP have been successfully applied to efficacy testing of disinfectants against biofilms formed by *Staphylococcus epidermidis* and *Pseudomonas aeruginosa*^16^ and antibiotic susceptibility testing against *Staphylococcus aureus* biofilms^17^. A crucial difference between DFR and MWP is the supply of nutrients. In the DFR, the nutrients flow over the glass slide to waste (it is a once-through system), while in the MWP, nutrients and cell by-products stay in the well for the length of the experiment and are mixed by swirling.

Previous studies have shown that biofilms grown under turbulent flow conditions display higher antimicrobial tolerance than those grown under laminar flow^18^,^19^,^20^ or in absence of flow^21^. Hence, the choice of the model has been found to influence the functional characteristics and architecture of biofilms, which can a have profound impact on the experimental outcome^21^. However, we are not aware of any comparative studies of efficacy testing in which biofilm reactors, such as DFR (with a fresh supply of nutrients), have been compared with MWP (in absence of a fresh supply of nutrients) with otherwise similar experimental conditions. Thus, the aim of this study was to evaluate the efficacy of clinically used antibiotics in preventing formation of *Staphylococcus aureus* biofilms using MWP and DFR. The antimicrobial tolerance of biofilms formed in both models was compared in order to gain insight into how choice of the model affects the experimental outcome of anti-biofilm efficacy assays.

## Results

### Selection of antibiotics for efficacy testing

Prior to the efficacy testing, an initial susceptibility testing of 27 antibiotics from several mechanistic classes was conducted using *S. aureus* ATCC 25923, a commonly used reference strain for antibiotic susceptibility testing as the model bacterium in MWP^22^. Serial two-fold dilutions of each antibiotic and exponentially grown *S. aureus* were simultaneously added into the wells. After 18 h, concentrations in which 90% inhibition of biofilm formation occurred, compared to the untreated controls were recorded as the minimum biofilm inhibitory concentrations (MBIC). MBIC refers to the concentration needed for a compound to cause 90% inhibitory effect on bacteria when a biofilm is developing^23^ and it is used instead of the minimum inhibitory concentration (MIC), which refers to the effect on planktonic bacteria (Supplementary Table S1). Effects of the antibiotics were also measured against pre-formed biofilms (Supplementary Table S2). For this purpose, biofilms were formed using exponentially grown *S. aureus* for 18 h, and thereafter, exposed to two-fold diluted series of antibiotics for 24 h. Based upon the MBIC values against *S. aureus* ATCC 25923, ten antibiotics with MBIC values lower or equal to 2 mg/L from four distinct mechanistic classes were selected for efficacy testing. Moreover, for confirmatory purposes, the selected antibiotics were tested against *S. aureus* Newman and *S. epidermidis* ATCC 35984 in MWP (Supplementary Tables S3 and S4).

### Comparison of anti-biofilm efficacies between MWP and DFR

Efficacy of the ten most active antibiotics was first assessed in MWP (Supplementary Table S5). Rifampicin, oxacillin and levofloxacin yielded a full log reduction (LR) at 100 μM (no countable colonies on TSA). Rifampicin was ranked as the most effective followed by three members of the β-lactam antibiotics (oxacillin, ampicillin and dicloxacillin), levofloxacin and doxycycline. Interestingly, rifampicin and all the β-lactam antibiotics caused a log reduction higher than 3 at a test concentration of 10 μM. However, levofloxacin reached a LR only of 0.8 when tested at 10 μM, while doxycycline yielded a higher LR of 2.3. Therefore, rifampicin, oxacillin (the most effective β-lactam) and doxycycline (instead of levofloxacin), from distinct mechanistic classes were chosen for the efficacy testing in the DFR.

**Figure 1:**
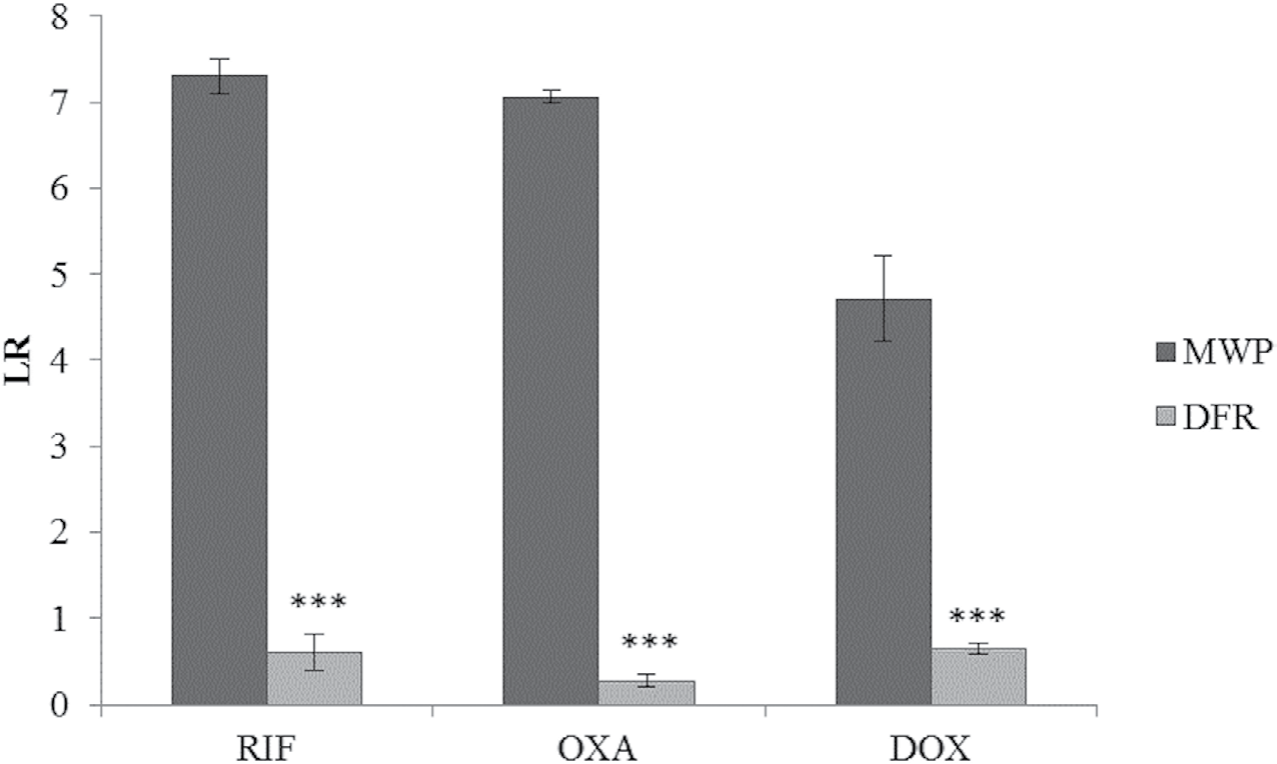
Log reduction (LR) for the most effective antibiotics when tested at 100 μM in MWP and DFR (under flow conditions). Results are shown as mean LR ± SD. RIF = rifampicin, OXA = oxacillin, DOX = doxycycline, MWP = microtiter well plate, DFR = drip flow reactor. *** - differences between the LR in both systems were statistically significant (*p* < 0.0001). Details of the number of replicates are presented in Table 1.

In the DFR, the efficacy of rifampicin and doxycycline against *S. aureus* ATCC 25923 was very similar, as reflected in the similar LR for both the 100 and 1000 μM treatments. In contrast, oxacillin was the least effective antibiotic. It did not display any efficacy in preventing biofilm formation, when tested at 1000 μM. Results from the efficacy testing of these three antibiotic in both models are summarized in Table 1. All antibiotics were significantly less effective in preventing biofilm formation in the DFR under flow conditions than in the MWP, as indicated by the consistently lower LR values (Fig. 1).

The rate of killing by antibiotics at 100 μM in DFR under flow conditions was lower (7 to 26 times) compared to MWP (Table 2). This analysis was made from the calculation of tolerance factors (TF). Tolerance factors, as described by Stewart^24^, were estimated on the basis of selected dose concentrations, duration and log reductions measured in both models according to the equation:


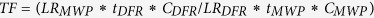


where *LR* refers to calculated log reduction in both model systems, *t* refers to dose duration (batch mode) in both models and *C* denotes antibiotic concentration applied to the testing. Considering the differences in LR as well as in TF, it can be concluded that the two models exhibited poor correlation here.

### Comparison of the MWP and DFR models

To shed light into the obtained results, various aspects of the two models were compared (Table 3). The surface-area-to-volume (SA/V) ratios were calculated for a single well in the MWP and one channel in the DFR and found to be in the same order of magnitude. In addition, the mean log density (LD) of control biofilms grown in both systems was calculated to exclude possible variations due to the bacterial concentrations. The log density of control biofilms grown in MWP ranged from 7.75 to 8.29 with a mean of 8.05 CFU/cm^2^, while for DFR biofilms this value ranged between 7.12 and 8.28 CFU/cm^2^ with a mean of 7.60 CFU/cm^2^. Thus, cell densities between the models were not substantially different, and in fact, DFR grown biofilms had an average log density that was 0.4 log units lower than in MWP. Therefore, this cannot explain the observed discrepancies. Mean colony forming units per milliliter (CFU/ml) of the planktonic inoculum used for biofilm formation in both models were also recorded and found not to be significantly different between MWP and DFR (*p* = 0.5235). Finally, a comparison of the statistical attributes calculated for the MWP and DFR was performed. The repeatability standard deviations calculated on the basis of the log densities (LD) of untreated, control biofilms were 0.17 and 0.41 for the MWP and DFR, respectively. Further, calculated Coefficients of Variation (CV) were low, and similar for both models. Small repeatability standard deviations and low CVs, calculated based on the untreated biofilm controls, indicate high repeatability between experiments in both models.

### Impact of nutrient flow

Rifampicin, oxacillin and doxycycline at 1000 and 100 μM were tested in DFR under static conditions. Results are shown in Table 4. All three antibiotics resulted in a full log reduction (no countable colonies on TSA after the overnight incubation at +37 °C) when tested at 1000 μM. Moreover, rifampicin and doxycycline yielded in a full log reduction also at 100 μM. Noteworthy, the cell density of the control biofilms grown in the absence of continuous nutrient flow in the DFR was observed to be significantly lower than of those grown under continuous flow of nutrients (6.46 ± 0.05 vs. 7.60 ± 0.41).

**Figure 2:**
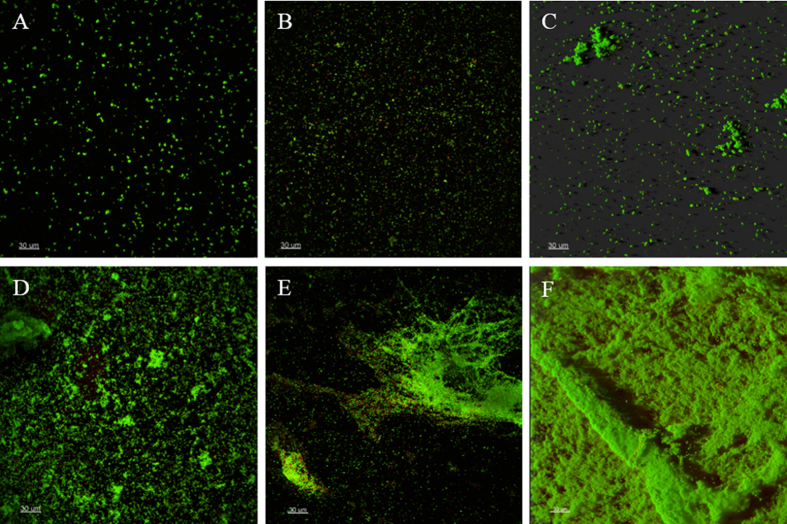
Confocal laser scanning microscopy images of *S. aureus* ATCC 25923 biofilms grown in MWP (**A**–**C**) and DFR (**D**–**F**). (**A**–**C**) are representatives taken from different locations of one well in a MWP and D-F from different locations within one coupon in DFR. Biofilms are stained with LIVE/DEAD BacLight kit. Scale bars correspond to 30 μM.

Tolerance factors were re-calculated to compare with the LRs obtained in the absence of flow in the DFR (Table 5). *S. aureus* ATCC 25923 biofilms were found to be equally tolerant to rifampicin and doxycycline at 100 μM (TF = 1), indicating no differences in the action of these antibiotics in MWP and DFR when the DFR was operated without a continuous flow of nutrients. However, even with no flow, the DFR grown biofilms were still more tolerant to oxacillin. Additionally, nutrient flow was shown to impact the biofilm architecture. Biofilms grown under flow conditions in DFR were observed to be fluffier than those grown in MWP. Moreover, different structural distribution of DFR grown biofilms was observed, when compared to MWP (Fig. 2).

Further, refreshment of the nutrient media in MWP during the incubation period of 24 h impacted the experimental outcome. All the tested antibiotics caused lower LR than in the initial testing in MWP (Table 6). Log density of the control biofilms formed on these conditions was 0.39 log higher than the control biofilms formed in MWP when the nutrient media was only added in the beginning of 24 h incubation (8.46 ± 0.04 vs. 8.07 ± 0.17).

## Discussion

*S. aureus* is responsible for a wide range of chronic and persistent biofilm infections and pose a serious threat to human health^6^. Once embedded in a biofilm, the phenotype and physiology of bacteria change profoundly, and they become highly tolerant to antimicrobials and host immune responses^25^. The efficacy of conventional antibiotics against biofilms is significantly reduced; concentrations needed to eradicate biofilm growing bacteria can be up to 1000-fold higher than therapeutic concentrations required for single-cells of the same species^24^,^26^. As biofilm infections are difficult to treat, prevention of biofilm formation is an important strategy in biofilm control, and antimicrobial therapy is considered as a viable option. Biofilm formation is initiated by reversible attachment of planktonic bacteria usually to a surface. At this stage, bacteria are still susceptible to antibiotics that support the usage of prophylactic antibiotic therapy for alloplastic surgery or early aggressive antibiotic therapy^4^,^27^. To capture the functional complexity of biofilms and the various conditions in which they can be formed, several experimental models have been proposed for efficacy testing of antimicrobials against biofilms^7^,^8^. However, in the literature, systematic comparison studies between models, under the same experimental conditions, are scarce.

Selection of antibiotics for efficacy testing was made based on an initial antibiotic susceptibility testing against *S. aureus* ATCC 25923 in MWP. To exclude strain-specific effects, MBIC values were also determined against *S. aureus* Newman and *S. epidermidis* ATCC 35984. Variability in MBIC values was observed for the test strains, whereas results from viability (resazurin) or biomass (crystal violet) quantification assays were overall similar. Rifampicin was the most effective antibiotic against all the test strains with the lowest MBIC values. Vancomycin in turn, appeared to be the least effective antibiotic for all conditions tested. Among the test strains, *S. aureus* ATCC 25923 was the most susceptible strain to the 10 selected antibiotics, with the lowest MBIC values. The effectiveness of the antibiotics was also tested against pre-formed (18 h) biofilms where a 50–60% reduction in biofilm viability was observed for the most effective antibiotics. None of antibiotics was able to decrease viability of existing biofilms by more than 70%, confirming that MWP is a suitable system to mimic the antimicrobial tolerance that has been extensively reported for bacterial biofilms^5^,^24^,^28^.

Based upon the results of efficacy testing in MWP, rifampicin, oxacillin and doxycycline as representatives of three distinct antibiotic classes were selected for efficacy testing performed in DFR. Rifampicin and oxacillin caused a full log reduction at 100 μM in MWP, while doxycycline produced a 4 log reduction. These three antibiotics were also effective at the lower concentration of 10 μM, displaying over 2 log reduction of the viable counts. However, when the DFR was operated with continuous flow of nutrients, none of these antibiotics was able to completely prevent biofilm formation even at the highest concentration (1000 μM). Overall, the efficacies of these antibiotics in the DFR (measured via LRs) were significantly lower than those found using MWP.

Rifampicin was the most effective antibiotic for the prevention of *S. aureus* biofilms when tested in MWP. In DFR, its efficacy at both test concentrations was comparable to doxycycline. Rifampicin is a fast acting bactericidal antibiotic that has been observed to be exceptionally effective against staphylococcal biofilms^29^. Rifampicin has been shown to prevent biofilm formation *in vitro* and it is also clinically effective in treatment of implant-associated staphylococcal infections. However, due to rapid emergence of resistant strains, rifampicin is recommended to be used in combination with other antibacterial agents. For instance, when used in combination with minocycline, daptomycin, and tigecycline as antibiotic lock therapy of vascular catheters, it has been shown to effectively eradicate even MRSA biofilms^30^. Doxycycline of tetracycline class is a bacteriostatic antibiotic that acts by inhibiting protein synthesis via 30 S ribosome and preventing binding of tRNA. Doxycycline has been reported to efficiently inhibit biofilm formation of *S. aureus in vitro*^31^. Another tetracycline, minocycline has been shown to reduce biofilm formation of *S. aureus* on the surfaces of central venous catheters^32^. Oxacillin displayed the lowest efficacy (lowest LR) when tested in DFR. Interestingly, when assayed at the highest test concentration, it produced a negative LR. Previous studies suggest that various antibiotics such as β-lactam antibiotics at sub-MIC concentrations have stimulatory effect on biofilm formation^6^,^33^. Here, on the contrary, the tested concentration was significantly higher than the determined MBIC value (0.52 μM). By using conductivity and light scattering techniques, oxacillin has been found to form aggregates in aqueous solutions^34^ and thus, it may become ineffective at particular concentrations, which may help explaining these results. Based on the results obtained here, it can also be concluded that anti-biofilm efficacy cannot be attributed to a particular mechanistic class of antibiotics.

Biofilm architecture, as well as antimicrobial tolerance of biofilms is dependent on growth conditions, such as hydrodynamics, nutrients and cell density^24^,^35^. Here, we attempted to analyse the possible factors that could affect the experimental outcome of the efficacy testing. The nutrient concentration, inoculum cell density and growth conditions were equal in the models during the one hour exposure time (batch phase) with the antibiotics. Furthermore, because there is no flow into or out of the MWP, we further evaluated the possibility that the higher LR obtained from MWP was a result of residual effects of remaining antibiotics in the wells. Thus, MWP trials with the three antibiotics at 100 μM were repeated by performing the exposures followed by two washing steps with sterile MQ-water to remove any remaining of the antibiotics, before adding fresh TSB for the incubation period. The washings did not remarkably impact the LR measured for the three antibiotics, excluding the possibility that a higher concentration of antibiotics would remain in the MWP when compared to DFR (Supplementary Table S6).

Moreover, the observed differences in LR values could not be attributed to differences in the cell density, differences in the planktonic inoculum concentration or surface-area-to-volume ratios. Both models provided reproducible data with repeatability standard deviations of ≤0.5, and CV of ≤5%. Thus, the continuous availability of fresh nutrients remained as the plausible explanation for the differences found in the LR values for the two models. In DFR, the biofilms were continuously supplied with fresh nutrient media, and the waste products and detached cells flowed out of the reactor. In contrast to DFR, there was no flow into or out from the well in MWP during the experiments, thus nutrient media added at the beginning along with waste products and dead cells stayed in the wells throughout the experiment^8^,^10^. Previous studies on flow dynamics in these two models have revealed that the wall shear present in both models is in the same order of magnitude; 0.3 Pa for DFR^36^ and under 1 Pa for MWP^37^, respectively and thus, differences in the wall shear do not seem to provide an adequate explanation for the registered differences in LR.

One of the major drawbacks of MWP is the nutrient depletion during incubation period. To overcome this issue, we further performed a variant of the initial efficacy test, in which the nutrient media was refreshed twice during the 24 h incubation. This impacted the measured efficacy of antibiotics. Doxycycline was the most effective antibiotic, while efficacy of rifampicin and oxacillin was remarkably reduced compared to the initial efficacy tests in MWP. The efficacy of the antibiotics was in all cases lower than in the initial testing in MWP, but still higher than in the DFR. Thus, it confirmed that refreshment of the media decreases the susceptibility of the formed biofilms to the antibiotics. These results are in line with our previous findings from DFR experiments. Rifampicin and oxacillin caused a full LR when tested in MWP without media refreshments and they were significantly less effective when the fresh nutrients were available in DFR.

Of note, when an incubation period of 24 h is applied to the efficacy testing in MWP, nutrient media is typically not refreshed during the experiments^38^,^39^,^40^,^41^,^42^. Thus, the most common experimental condition is the one initially presented here, where no media refreshments are performed. In case of longer incubation periods, refreshment of media is typically done only after 24 h^6^,^17^,^43^,^44^.

The impact of nutrient flow was then subsequently demonstrated by measuring efficacy without flow in DFR. All the antibiotics tested resulted in a full LR when tested at 1000 μM, and the rate of killing by rifampicin and doxycycline at 100 μM was equal to MWP-grown biofilms. Of note, the mean LD of control biofilms grown in static conditions in DFR was lower (~1 log unit) than of those grown under continuous flow in the DFR. Moreover, biofilms grown under continuous flow (DFR) exhibited different architecture than those grown in the closed system (MWP). More variability in biofilm structure and heterogeneity of biofilms was observed. Such features are also present in biofilms occurring *in vivo*^45^. This structural heterogeneity can also have an impact on antibiotic efficacy if antibiotics do not diffuse evenly across a biofilm in DFR.

Even though both models can be reproducibly utilized for efficacy testing, they only partially reflect *in vivo* conditions, especially those occurring in chronic infections^46^. For instance, host defence mechanisms are lacking in these models and further, biofilm structures *in vivo* differ from *in vitro* structures in terms of physical dimensions and microenvironments^45^. Both models have also their benefits and drawbacks. MWP allow high throughput and testing of large chemical libraries, using small volumes of antimicrobials and minor media consumption. Testing in MWP can also be regarded as user-friendly because no specific instrumentation is needed. However, growth conditions vary during the experiments due to nutrient depletion. Moreover, there is no flow within the system and thus, it does not allow for the formation of biofilms that are typically found *in vivo*^8^. On the other hand, hydrodynamic conditions within the MWP can be still adjusted to be similar to those occurring in various biomedical settings *in vivo* by changing the shaking frequency^47^. In turn, DFR is more laborious and larger volumes of media and test samples are needed. Moreover, only few antimicrobials (usually 4–6) can be tested in parallel. However, constant nutrient flow, as well as bypass of waste products and dead cells, creates a uniform environment for biofilm growth^7^. For example, the flow rate (0.82 mL/min) used in this study, mimics the flow rate present in urinary tract catheters (40–80 mL/h)^47^.

To the best of our knowledge, the present contribution represents the first comparative study in which efficacy testing of antibiotics has been conducted under the same experimental conditions in two distinct models characterized by the absence and presence of flow, respectively. Our results revealed that biofilms grown in DFR under continuous flow of nutrients displayed higher antimicrobial tolerance than those grown without flow. These findings further support previous suggestions that the choice of biofilm model system can have a considerable impact on experimental outcome^8^,^45^,^48^. Altogether, this contribution highlights the importance of evaluating the *in vitro* anti-biofilm efficacy in different types of biofilm models, under conditions as close to the clinical setting as possible to facilitate the selection of the best compounds.

## Methods

### Antibiotics

A group of 27 antibiotics belonging to different mechanistic classes was initially explored (Supplementary Table S7). Stock solutions were prepared in Mueller Hinton Broth (MHB, Fluka Biochemika, Buchs, Switzerland) except for ampicillin, clindamycin, doxycycline and rifampicin, which were dissolved in dimethyl sulfoxide (DMSO, Sigma-Aldrich, St. Louis, MO, US).

### **Bacterial strains and growth conditions**

Biofilm forming control strains of *Staphylococcus aureus* (ATCC 25923 and Newman, both clinical) and *Staphylococcus epidermidis* (ATCC 35984, clinical) were stored at −70 °C as cryogenic stocks and plated on tryptic soy agar (TSA, Fluka Biochemika, Buchs/Becton Dickinson & Company, Sparks, MD, US). For the inoculum, colonies were collected from the TSA plates using a 1 μL inoculation loop and added into 3 mL of 30 g/L TSB. Bacteria were grown in aerobic conditions at 37 °C, 220 rpm for 16–18 h. Overnight cultures were diluted in TSB (*S. aureus* 1:1000 and *S. epidermidis* 1:100), and incubated at 37 °C, 200 rpm until an OD_595_ of 0.3–0.5 corresponding to 10^8^ colony forming units per milliliter (CFU/mL). The viable cell density was confirmed by serially diluting and plating on TSA.

### Antibiotic susceptibility assays

The minimum biofilm inhibitory concentrations (MBIC) of antibiotics were determined against *S. aureus* (2 strains) and *S. epidermidis* (one strain)^49^. Antibiotics were diluted in TSB and tested in two replicate wells (technical replicates), three times (three biological replicates) using two-fold dilutions with concentrations ranging from 4.88 × 10^−4^ mg/L to 1024 mg/L. For MBIC measurements, 5 μL of antibiotics and 195 μL of bacterial culture (10^6^ CFU/mL) in 30 g/L TSB were added simultaneously to 96-microtiter well plates (Nunclon™ Δ Nunc surface, Thermo Fischer Scientific, Roskilde, Denmark) and incubated at 37 °C, 200 rpm for 18 h. Effects of the antibiotics were quantified on the basis of viability and biomass using resazurin and crystal violet staining assays, respectively, as in refs 50 and 51. First, a solution (200 μL) of 20 μM resazurin (Sigma-Aldrich, St. Louis, MO, USA), prepared in phosphate buffered saline (PBS, Lonza, Verviers, Belgium) was added to each well. Plates were incubated in darkness, 200 rpm, at room temperature (RT), for 20 minutes and fluorescence was detected using a Varioskan LUX reader (Thermo Scientific, Vantaa, Finland) at λ_ex_ = 560 nm; λ_em_ = 590 nm. Subsequently, the resazurin stain was removed using a multichannel pipette and biofilms were stained with 200 μL of 2.3% (w/v) crystal violet (Sigma-Aldrich Co, St. Louis, MO, USA) and washed three times with MQ water. Remaining stain was dissolved by adding 200 μL of 96% (v/v) ethanol per well and the plates were incubated at RT for 1 h before measuring the absorbance (λ = 595 nm). Additionally, effects of the antibiotics were measured on pre-formed biofilms, which were formed from diluted cultures in TSB (10^6^ CFU/mL, 200 μL per well, 37 °C, 200 rpm) for 18 h. After 18 h, the planktonic phase was removed, antibiotics and fresh media were added and plates were incubated for 24 h (37 °C, 200 rpm). Biofilms were stained with resazurin as described above.

### Efficacy testing

**Figure 3:**
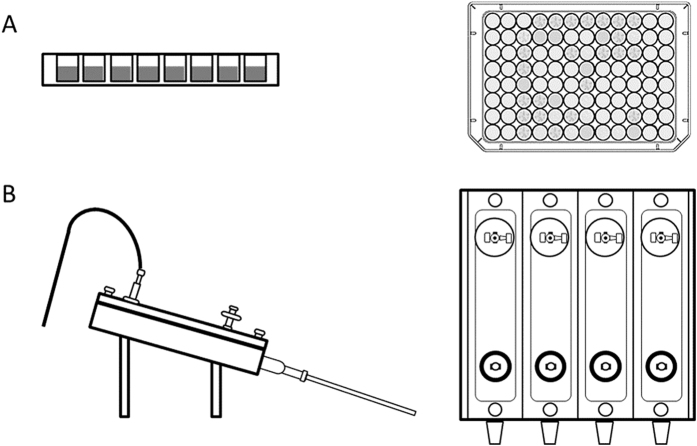
Schematic representation of models applied to the efficacy testing. In (**A**) a 96-Microtiter well plate (MWP) is shown while in (**B**) a Drip Flow Reactor (DFR) is depicted. In both cases, the left images correspond to side views, while the right images correspond to top views.

An experimental set-up comprising a batch phase (exposure to antibiotics for one hour) followed by incubation or continuous flow phase of 24 hours was employed in efficacy testing of selected antibiotics in MWP and DFR (Fig. 3). In MWP, the efficacy of 10 antibiotics was tested at 0.1, 1, 10 and 100 μM in duplicates, in at least three biological rounds (biological replicates). Based upon these results, three antibiotics were selected for DFR studies, in which these antibiotics were tested at 100 and 1000 μM at least as five biological replicates (in each reactor experiment only 4 samples can be included at once, see Fig. 3). Biofilms were formed using a diluted culture of *S. aureus* ATCC 25923 (10^7^ CFU/mL in TSB). Both models are detailed below.

In MWP, 4 μL of the 10 antibiotics and 196 μL of the *S. aureus* culture were simultaneously added to the wells. Control wells containing 200 μL of bacteria and cell-free wells with 200 μL of TSB were included. After the contact time (1 h, 37 ± 1 °C, 200 rpm), the planktonic suspension was removed from the wells and replaced with 200 μL of fresh TSB (30 g/L). The plates were incubated at 37 °C, 200 rpm, for an additional 24 h. At the end of the 25 hours, the planktonic phase was discarded and biofilms were quantified as in ref. 52. Biofilms were scraped off the wells into 100 μL of TSB using sterile plastic inoculation loops. The wells were rinsed with additional 100 μL of TSB, and the two suspensions were combined in Eppendorf tubes. The tubes were sonicated at 25 °C, 35 kHz for 5 min (Bandelin Sonorex Digitec, Zurich, Switzerland) followed by serial dilution and plating on TSA. The colony forming units (CFU) were counted after overnight incubation at 37 ± 1 °C. In addition, to exclude the possible occurrence of residual effects due to remaining antibiotics in the MWP, exposure to the three selected antibiotics in this model was repeated with some modifications. After removal of the planktonic phase, wells were washed twice with sterile MQ-water (200 μL) before adding fresh TSB to the wells. The plates were then incubated at 37 °C, 200 rpm, for an additional 24 h, and the biofilms were quantified in the same manner as described above. Finally, experiments with the three selected antibiotics in MWP were performed according to the initial protocol but the nutrient media was refreshed twice during the 24 h incubation period. The media was pipetted out from the wells and fresh nutrient media was added after 8 and 17 hours.

In the DFR, a suspended culture of *S. aureus* and antibiotics (rifampicin, oxacillin or doxycycline) were premixed (15 mL suspensions) and added to three of the reactor channels, while 15 mL of bacteria was added to the fourth channel. Biofilms were formed by operating the reactor (model DFR 110-4, BioSurface Technologies Corporation, Bozeman, MT, USA) in batch mode (no flow) at 35 ± 2 °C for 1 h. The reactor was then drained and operated in continuous flow mode (reactor slope 10°) with a flow rate of 0.82 mL/min/channel of 3 g/L TSB for 24 h. At the end of the 25 hours, coupons were removed from the channels and rinsed by gently immersing the slide containing the biofilm in 45 mL of sterile dilution water. Biofilms were scraped off the coupons into 35 mL of 0.5% (w/v) Tween 80 (Fischer Scientific, Fair Lawn, NJ, US) prepared in TSB (30 g/L). The surface of the coupons was rinsed five times with 1 mL of 0.5% (w/v) Tween 80 prepared in TSB. Biofilms were quantified as in ref. 10. Aggregates were vortexed for 30 s, sonicated for 30 s (Elma transsonic, GmbH & Co, Germany, 45 kHz, 25 °C) in 2 cycles and vortexed for 30 s one more time. Samples were serially diluted and plated on TSA and viable colonies were counted after overnight incubation at 36 ± 2 °C. Additionally, efficacy testing was conducted in the absence of flow following the protocol described above with one modification: after the one hour contact time, the reactor was drained, and 15 ml of fresh nutrient media (TSB 30 g/L) was added into the channels. The reactor was incubated in a level position (without flow) at 35 ± 2 °C for 24 h.

### Confocal Scanning Laser Microscopy imaging

For imaging, biofilms were formed in polystyrene 12-microtiter well plates (flat-bottom, Becton Dickinson & Company, Franklin Lakes, NJ, US) and in DFR. Briefly, 1.5 mL of *S. aureus* ATCC 25923 culture (10^7^ CFU/ml) was added per well and the MWP were incubated at 37 °C (200 rpm) for 1 h. Thereafter, the bacterial suspensions were discarded and 1.5 mL of TSB (30 g/l) was added into the wells, and the plates were incubated for additional 24 h (37 °C, 200 rpm). In DFR, biofilms were formed as untreated control biofilms (described above). Microscopic imaging was performed on an upright Leica SP5 Confocal Scanning Laser Microscope using the 488 and 561 nm laser excitation lines. Biofilms were stained with LIVE/DEAD *Bac*Light Bacterial Viability Kit (Invitrogen #L7012, Carlsbad, CA, US) for 20 minutes, rinsed, and then imaged in a fully-hydrated state using extra-long working distance water immersion objectives. Images were processed using Imaris x64 8.0.1 software (Bitplane Scientific Software, Zurich, Switzerland).

### Statistical analysis

In the efficacy testing, all calculations were conducted using the log density (LD) values as described in refs 10 and 52 for the MWP and DFR, respectively. Anti-biofilm efficacy of each treatment was estimated by calculating Log reduction (LR) from the difference of the mean log_10_ density of untreated (control) and antibiotic treated biofilms (CFU/cm^2^)^16^. Substitution rule was applied to the calculations of LR. Artificial counts of 0.1 for the observed zero on one plate at the first counted dilution were used for the calculations. Assay repeatability was estimated by calculating standard deviations for all the mean values, and assay performance was further evaluated by means of the Coefficient of Variation (CV)^53^. Statistical analysis was performed using GraphPad Software (Prism, version 5.0 for Mac). For paired comparisons of the data, an unpaired *t*-test with Welch’s correlation was utilized (*p* < 0.05 was considered statistically significant).

## Additional Information

**How to cite this article:** Manner, S. *et al*. Prevention of *Staphylococcus aureus* biofilm formation by antibiotics in 96-Microtiter Well Plates and Drip Flow Reactors: critical factors influencing outcomes. *Sci. Rep.* **7**, 43854; doi: 10.1038/srep43854 (2017).

**Publisher's note:** Springer Nature remains neutral with regard to jurisdictional claims in published maps and institutional affiliations.

## Supplementary Material

Supplementary Information

## Figures and Tables

**Table 1:** Summary of results (mean ± SD) for LR obtained with selected antibiotics in MWP and DFR (under flow conditions).

Model	Effect (LR ± SD) of antibiotics			
	Test concentration (μM)	RIF	OXA	DOX
Microtiter well plates (MWP)	**0.1**	0.36 ± 0.11	0.35 ± 0.09	0.05 ± 0.19
		n = 6	n = 6	n = 6
	**1**	3.90 ± 0.77	1.39 ± 0.33	0.40 ± 0.38
		n = 6	n = 6	n = 6
	**10**	5.45 ± 0.27	5.42 ± 0.29	2.46 ± 0.24
		n = 6	n = 6	n = 6
	**100**	7.31 ± 0.20	7.20 ± 0.21	4.68 ± 0.40
		n = 6	n = 6	n = 6
Drip flow reactor (DFR)	**100**	0.61 ± 0.21	0.27 ± 0.07	0.65 ± 0.07
		n = 8	n = 5	n = 5
	**1000**	1.51 ± 0.18	−0.18 ± 0.34	1.48 ± 0.27
		n = 7	n = 5	n = 5

**Table 2:** Tolerance factors (TF) calculated based on the LR at 100 μM obtained in MWP and DFR (under flow conditions).

Antibiotic	Tolerance Factor (TF)
Rifampicin	12
Oxacillin	26
Doxycycline	7

**Table 3:** Comparison of surface-area-to-volume (SA/V) ratios, log density of control biofilms, planktonic inoculum concentrations and statistical parameters between MWP and DFR.

	Calculated surface-area-to-volume (SA/V) ratio	Control biofilm (log_10_ CFU/cm^2^) mean ± SD	Planktonic inoculum (log_10_ CFU/mL) mean ± SD	Repeatability Standard deviation (SD)	Coefficient of variation (CV) (%)
MWP	7.961 cm^−1^	8.07 ± 0.17	8.29 ± 0.21	0.17	2
		n = 27	n = 23		
DFR	2.708 cm^−1^	7.60 ± 0.41	8.26 ± 0.25	0.41	5
		n = 11	n = 13		

**Table 4:** Log reduction (LR) for the three selected antibiotics when tested at 100 μM and 1000 μM in DFR (under static conditions). Antibiotics were tested once, except for rifampicin at 1000 μM twice.

Antibiotic	Effect (LR) of antibiotics	
	Test concentration (μM)	
	100	1000
Rifampicin	5.43	5.48
Oxacillin	1.20	5.48
Doxycycline	5.43	5.48

**Table 5:** Tolerance factors calculated using results from static experiment in DFR.

Antibiotic	Tolerance Factor (TF)
Rifampicin	1
Oxacillin	6
Doxycycline	1

**Table 6:** Log reductions (LRs) obtained with the three selected antibiotics when tested at 100 μM in MWP and nutrient media was refreshed twice during the incubation period.

Antibiotic	Effect (LR ± SD) of antibiotics
	Test concentration (μM) 100
Rifampicin	4.32 ± 0.08
Oxacillin	2.55 ± 0.06
Doxycycline	4.91 ± 0.03

Antibiotics were tested in duplicates, in three separate experiments.

The mean log density of untreated control biofilms was 8.46 ± 0.04.
