# Supplementary material for: Prevention of Staphylococcus aureus biofilm formation by antibiotics in 96-Microtiter Well Plates and Drip Flow Reactors: critical factors influencing outcomes
Source: Sci Rep. 2017 Mar 2;7:43854. doi: 10.1038/srep43854 (PMC5333151; doi:10.1038/srep43854)
Supplement: Supplementary Information [file srep43854-s1.pdf]

Prevention of *Staphylococcus aureus* biofilm formation by antibiotics in 96-Microtiter Well Plates and Drip Flow Reactors: critical factors influencing outcomes

Suvi Manner, Darla M Goeres, Malena Skogman, Pia Vuorela, Adyary Fallarero

Supplementary information

Supplementary table S1. MBIC values determined on *S. aureus* ATCC 25923 by resazurin and crystal violet staining, respectively. The selected antibiotics are marked in bold. Antibiotics were tested in two replicates, three times (three biological replicates).

Antibiotic	MBIC			
	Viability		Biomass	
	mg/L	μM	mg/L	μM
**Penicillin G**	0.0156	0.04	N/A	N/A
**Clindamycin**	0.0313	0.07	0.0313	0.07
**Rifampicin**	0.0313	0.04	0.0625	0.08
**Doxycycline**	0.125	0.12	0.25	0.24
**Tetracycline**	0.25	0.52	0.125	0.26
**Oxacillin**	0.25	0.52	0.125	0.30
**Ampicillin**	0.25	0.62	0.125	0.31
**Dicloxacillin**	0.5	0.98	0.5	0.98
**Levofloxacin**	0.5	1.38	0.5	1.38
**Vancomycin**	2	1.37	N/A	N/A
Fusidic acid	2	3.71	2	3.71
Linezolid	2	5.93	2	5.93
Ciprofloxacin	4	12.07	2	6.04
Erythromycin	4	5.46	2	2.73
Tobramycin	8	17.11	4	8.56
Trimethoprim	8	27.56	4	13.78
Teicoplanin	8	4.26	8	4.26
Azithromycin	8	10.68	8	10.68
Kanamycin	16	27.46	16	27.46
Neomycin	32	35.21	16	17.60
Streptomycin	32	43.91	N/A	N/A
Polymyxin B	64	46.19	32	23.09
Chloramphenicol	128	396.16	128	396.16
Daptomycin	1024	631.83	512	315.91
Amphotericin B	NO	NO	NO	NO
Gramicidin A	NO	NO	NO	NO
Metronidazole	NO	NO	NO	NO

N/A = not available

NO = no inhibition

Supplementary table S2. Effects on pre-formed *S. aureus* ATCC 25923 biofilms using resazurin staining assay. Antibiotics were tested in two replicates, three times (three biological replicates).

Antibiotic	Effects on pre-formed biofilms		
	Viability		Inhibition-%
	mg/L	µM	
Rifampicin	⩾0.0313	0.04	~50
Penicillin G	⩾0.0625	0.18	~60
Dicloxacillin	⩾0.125	0.24	~50
Oxacillin	0.125	0.30	~50
Clindamycin	⩾0.25	0.59	~50
Ampicillin	⩾0.5	1.24	~60
Fusidic acid	⩾1	1.86	~60
Tetracycline	⩾2	4.16	~50
Doxycycline	⩾4	3.90	~60
Vancomycin	⩾8	5.38	~50
Linezolid	⩾16	47.42	~60
Ciprofloxacin	⩾16	48.29	~60
Tobramycin	⩾16	34.22	~50
Teicoplanin	⩾32	17.04	~60
Neomycin	⩾32	35.21	~50
Erythromycin	⩾32	43.60	~60
Kanamycin	⩾32	54.93	~60
Levofloxacin	⩾32	88.55	~50
Chloramphenicol	⩾256	792.32	~70
Polymyxin B	⩾512	369.51	~60
Amphotericin B	NO	NO	-
Azithromycin	NO	NO	-
Daptomycin	NO	NO	-
Gramicidin A	NO	NO	-
Metronidazole	NO	NO	-
Streptomycin	NO	NO	-
Trimethoprim	NO	NO	-

NO = no inhibition

Supplementary table S3. MBIC values of the top antibiotics determined on *S. aureus* Newman and effects on pre-formed biofilms using resazurin and crystal violet staining assays, respectively. Antibiotics were tested in two replicates, three times (three biological replicates).

Antibiotic	MBIC				Effects on pre-formed biofilms		
	Viability		Biomass		Viability		Inhibition-%
	mg/L	μM	mg/L	μM	mg/L	μM	
Clindamycin	0.0313	0.07	0.0625	0.15	⩾1	2.35	~70
Dicloxacillin	0.0625	0.12	0.0625	0.12	⩾1	1.96	~70
Rifampicin	0.125	0.15	0.125	0.15	⩾0.0313	0.04	~60
Penicillin	0.125	0.35	0.125	0.35	⩾16	44.90	~60
Doxycycline	0.25	0.24	0.25	0.24	⩾1	0.97	~70
Levofloxacin	0.25	0.69	0.25	0.69	⩾32	88.55	~50
Oxacillin	0.5	1.18	0.5	1.18	⩾16	37.79	~70
Ampicillin	0.5	1.24	0.5	1.24	⩾16	39.66	~60
Tetracycline	1	2.08	1	2.08	⩾8	16.64	~50
Vancomycin	32	21.54	32	21.54	⩾128	86.15	~70

Supplementary table S4. MBIC values of the top antibiotics determined on *S. epidermidis* ATCC 35984 and effects on pre-formed biofilms using resazurin and crystal violet staining assays, respectively. Antibiotics were three times (three biological replicates).

Antibiotic	MBIC				Effects on pre-formed biofilms		
	Viability		Biomass		Viability		Inhibition-%
	mg/L	µM	mg/L	µM	mg/L	µM	
Rifampicin	0.0156	0.02	0.0156	0.02	⩾0.0313	0.04	~50
Penicillin	0.125	0.35	0.125	0.35	⩾0.125	0.35	~55
Dicloxacillin	0.25	0.49	0.25	0.49	⩾1	1.96	~50
Clindamycin	0.25	0.59	0.25	0.59	⩾2	4.71	~55
Oxacillin	0.5	1.18	0.5	1.18	⩾1	2.36	~55
Doxycycline	0.5	0.49	0.5	0.49	⩾1	0.97	~55
Ampicillin	0.5	1.24	0.5	1.24	⩾2	4.96	~55
Levofloxacin	0.5	1.38	0.5	1.38	⩾32	88.55	~40
Tetracycline	2	4.16	2	4.16	⩾2	4.16	~65
Vancomycin	16	10.77	16	10.77	⩾64	43.08	~40

Supplementary table S5. Summary of results reported as log reductions (LR) (mean ± SD) obtained with each test concentration of tested antibiotics in MWP. All the antibiotics were tested in duplicates at least three times (three biological replicates).

Antibiotic	Effect (LR ± SD) of antibiotics			
	Test concentration (μM)			
	0.1	1	10	100
Rifampicin	0.36 ± 0.09	3.90 ± 0.77	5.45 ± 0.27	7.31 ± 0.20
Oxacillin	0.35 ± 0.09	1.39 ± 0.33	5.42 ± 0.28	7.20 ± 0.21
Ampicillin	0.15 ± 0.14	0.78 ± 0.19	5.02 ± 0.29	5.30 ± 0.14
Dicloxacillin	0.09 ± 0.17	0.84 ± 0.04	3.90 ± 0.17	5.54 ± 0.40
Levofloxacin	0.00 ± 0.17	0.19 ± 0.10	0.82 ± 0.12	6.89 ± 0.05
Doxycycline	0.05 ± 0.19	0.40 ± 0.38	2.46 ± 0.24	4.68 ± 0.40
Clindamycin	-0.11 ± 0.14	0.02 ± 0.09	3.95 ± 0.18	4.34 ± 0.17
Tetracycline	0.17 ± 0.15	0.31 ± 0.15	0.83 ± 0.21	4.19 ± 0.26
Penicillin G	0.09 ± 0.05	1.54 ± 0.53	1.90 ± 0.21	2.91 ± 0.22
Vancomycin	0.09 ± 0.04	0.27 ± 0.08	0.35 ± 0.11	0.59 ± 0.11

Supplementary table S6. Summary of results reported as log reductions (LR) (mean ± SD) obtained with selected antibiotics at 100 μM in MWP, when washing step was included. Antibiotics were tested in duplicates, three times.

Antibiotic	Effect (LR) of antibiotics
	Test concentration (μM) 100
Rifampicin	6.85 ± 0.16
Oxacillin	6.87 ± 0.15
Doxycycline	4.04 ± 0.38

The mean log density of untreated control biofilms was 7.84 ± 0.12.

Supplementary table S7. List of antibiotics included in this study.

Antibiotic	Supplier	Class	Mechanism of action
Ampicillin	Sigma-Aldrich Co, St. Louis, MO, US	β-lactam	Inhibition of cell wall biosynthesis
Dicloxacillin sodium salt monohydrate	Sigma-Aldrich Co, St. Louis, MO, US	β-lactam	
Oxacillin sodium salt	Sigma-Aldrich Co, St. Louis, MO, US	β-lactam	
Penicillin G sodium salt	Sigma-Aldrich Co, St. Louis, MO, US	β-lactam	
Polymyxin B	Sigma-Aldrich Co, St. Louis, MO, US	Polymyxin	
Teicoplanin	Cayman chemicals, Ann Arbor, MI, US	Glycopeptide	
Vancomycin	Sigma-Aldrich Co, St. Louis, MO, US	Glycopeptide	
Doxycycline hyclate	Cayman chemicals, Ann Arbor, MI, US	Tetracycline	Protein synthesis inhibition
Tetracycline	Cayman chemicals, Ann Arbor, MI, US	Tetracycline	
Kanamycin	Sigma-Aldrich Co, St. Louis, MO, US	Aminoglycoside	
Neomycin	Sigma-Aldrich Co, St. Louis, MO, US	Aminoglycoside	
Streptomycin	Sigma-Aldrich Co, St. Louis, MO, US	Aminoglycoside	
Tobramycin	Sigma-Aldrich Co, St. Louis, MO, US	Aminoglycoside	
Azithromycin	Cayman chemicals, Ann Arbor, MI, US	Macrolide	
Erythromycin	Sigma-Aldrich Co, St. Louis, MO, US	Macrolide	
Chloramphenicol	Sigma-Aldrich Co, St. Louis, MO, US	Lincosamide	
Clindamycin	Cayman chemicals, Ann Arbor, MI, US	Lincosamide	
Linezolid	Cayman chemicals, Ann Arbor, MI, US	Oxazolidinone	
Fusidic acid	Sigma-Aldrich Co, St. Louis, MO, US	Fucidane	
Trimethoprim	Sigma-Aldrich Co, St. Louis, MO, US	Dihydrofolate reductase inhibitor	Folic acid synthesis inhibition
Ciprofloxacin	Sigma-Aldrich Co, St. Louis, MO, US	Fluoroquinolone	DNA synthesis inhibition
Levofloxacin	Sigma-Aldrich Co, St. Louis, MO, US	Fluoroquinolone	
Metronidazole	Sigma-Aldrich Co, St. Louis, MO, US	Nitroimidazole	

Rifampicin	Sigma-Aldrich Co, St. Louis, MO, US	Rifamycin	RNA synthesis inhibition
Amphotericin B	Sigma-Aldrich Co, St. Louis, MO, US	Polyene	Targets the cell membrane
Daptomycin	Cayman chemicals, Ann Arbor, MI, US	Lipopeptide	
Gramicidin A	Sigma-Aldrich Co, St. Louis, MO, US	Peptide	
